# Notes and Comments

**Published:** 1894-01

**Authors:** 


### Notes and Comments.[1]

Excellence.—The end and aim of human life is manly excellence. That consists in development of body and spirit; all else is means; the formation of a manly character is the end. He makes the most of life here who becomes most of a man, does the most of human duties, and so has the largest quantity and highest quality of manhood.

---

Stimulants and Hemorrhage.—Never give stimulants in a case of severe hemorrage. The faint feeling or irresistible inclination to lie down is nature's own method of circumventing the danger by quieting the circulation and lessening the expulsive force of the heart, thus favoring the formation of clot at the site of injury.—*Clinique.*

---

How to Rest.—"To understand the way to rest is of more importance than to know how to work. The latter can be learned easily; the former it takes years to learn, and some people never learn the art of resting. It is simply a change of scenes and activities. Loafing may not be resting. Sitting down for days with nothing to do is not restful. A change is needed to bring into play a different set of faculties, and to turn the life into a new channel. The man who works hard finds his best rest in playing hard. The man who is burdened with care finds relief in something that is active, yet free from responsibility. Above all, keep good-natured, and don't abuse your best friend, the stomach."

---

Professional Fees.—Almost the first question a young practitioner asks is, "What shall I charge?" "What do you charge?" These two questions many times have caused him years of sorrow and hopeless longing for the clientele that never comes.

If he had commenced with the idea of doing a great deal of work

[1] The assistant editor solicits contributions for this department,—new methods, new remedies and formulas, or any short practical note which may prove of value to the practitioner or student. Address 1506 Arch Street, Philadelphia.

for a little money, his skill would have increased, his experience enlarged, his reputation spread. Patients would have flocked to him as to the one person whom they would permit to fill their teeth, and then, independent of the whims and idiosyncrasies of the few, he could have charged fair prices with a feeling of security in the rapid development of his reputation and the enlargement of his bank account.—*Dr. Truman.*

